# Mild COVID-19 Was Not Associated with Impaired IVF Outcomes or Early Pregnancy Loss in IVF Patients

**DOI:** 10.3390/jcm11185265

**Published:** 2022-09-06

**Authors:** Yossef Kabalkin, Yaakov Bentov, Moran Gil, Ofer Beharier, Sireen Jaber, Arbel Moav-Zafrir, Dua’ Khwies, Assaf Ben-Meir, Efrat Esh Broder, Asnat Walfisch, Hananel E. G. Holzer, Anat Hershko Klement

**Affiliations:** 1 Faculty of Medicine, Hebrew University of Jerusalem, Jerusalem 9112102, Israel; 2 Division of Obstetrics and Gynecology, Hadassah-Hebrew University Medical Center, Jerusalem 9112102, Israel; 3 The IVF Unit, Hadassah Mount Scopus-Hebrew University Medical Center, Jerusalem 91240, Israel; 4 The IVF Unit, Hadassah Ein Kerem-Hebrew University Medical Center, Jerusalem 91220, Israel

**Keywords:** COVID-19, SARS-CoV-2, in vitro fertilization, infertility, pregnancy outcomes

## Abstract

Data collection regarding the effects of COVID-19 on reproduction is ongoing. This study examined the effect of COVID-19 on IVF cycle parameters and early pregnancy outcomes. It included two arms: the first compared non-exposed cycles to post-SARS-CoV-2 IVF cycles. Sperm parameters were also compared. The second, prospective arm compared pregnancy outcomes among IVF patients who contracted COVID-19 during early pregnancy to those who did not. None of the patients were vaccinated against SARS-CoV-2. The first arm included 60 treatment cycles of women with confirmed COVID-19, compared to 60 non-exposed cycles (either the same patient before exposure or matched non-exposed patients). The outcomes of the treatment cycles did not differ significantly between exposed and non-exposed groups, including number of oocytes, endometrial thickness, fertilization rate and number of top-quality embryos. In 11 cycles, the male partner had also recently recovered: sperm concentration was lower post-exposure: 6.27 million/mL vs. 16.5 pre-exposure (*p* = 0.008). In 189 patients with IVF-achieved pregnancies, pregnancy loss and hospital admissions did not differ between exposed and non-exposed groups. IVF treatment outcomes and the rate of early pregnancy loss appears to be unaffected by SARS-CoV-2 disease, despite a minor decline in sperm concentration among recent recoverees.

## 1. Introduction

By the end of 2019, the coronavirus disease 2019 (COVID-19) had rapidly spread to become a global pandemic affecting more than half a billion people and resulting in approximately six million fatalities worldwide. Although more than two years have passed since the outbreak, the disease has not yet disappeared. Long-term effects of the virus have not been fully studied and new variants are still emerging. The multisystemic disease has short- and long-term effects that may involve any organ system [1,2]. However, evidence-based data on potential effects on male and female reproduction are still not fully understood.

Studies have shown that SARS-CoV-2 invades cells mainly through binding to the angiotensin-converting enzyme 2 receptor and is activated by transmembrane serine protease 2. A reproductive co-expression of these two membranous proteins that is necessary for SARS-CoV-2 cell invasion has not been detected; therefore, it has been assumed that human reproductive organs are less susceptible to direct effects from the virus [3,4,5]. However, several studies have detected high ACE-2 expression levels in testicular cells, predominantly in spermatogonia, seminiferous duct cells, Sertoli cells and Leydig cells [6], which may indicate vulnerability of the testicles to SARS-CoV-2 infection. Regarding the question of transmission of SARS-CoV-2 via sperm, a systematic review found that the likelihood of SARS-CoV-2 in the semen of COVID-19 patients is very small [7]. Another study [8] analyzed semen samples within 24 h of a positive nasopharyngeal swab; 1/32 samples were found to be positive for SARS-CoV-2 RNA. However, the authors commented that oral contamination during sample production could not be ruled out. Best et al. [9] analyzed semen samples from recovered men that were obtained 11–64 days after testing positive for SAR-CoV-2 infection. In this study, no viral RNA was detected in any of the samples.

Regarding the possibility of an indirect effect of the virus, one study [10] reported a negative correlation between anti-SARS-CoV-2 IgG in follicular fluid and oocyte and mature oocyte yields, while another did not find a correlation [11]. In addition, acute COVID-19 is often associated with systemic inflammation and fever, which may impact the function of the reproductive system indirectly [12,13,14]. Furthermore, in affected men, there have been reports of higher rates of orchitis and impaired spermatogenesis. However, these were early, anecdotal reports among men with severe COVID-19 [15].

Regarding risks that COVID-19 may pose during pregnancy, it has been reported that pregnant women who contract the virus are more likely to require intensive care admission, mechanical ventilation, and extracorporeal membrane oxygenation, as well as increased mortality rates [16,17]. Some of the risk-factors among pregnant women for severe complications related to COVID-19 are age over 25 years, pre-pregnancy obesity, chronic hypertension, and pre-pregnancy diabetes [18]. These risk-factors are significantly more common among patients who conceived following in vitro fertilization (IVF) [19]. In addition, it has been reported that COVID-19 is associated with a higher risk for thromboembolism [20], in addition to the risk imposed by IVF treatment [21]. Therefore, the combination of the risk factors characteristic of this population together with IVF treatment may put IVF patients with COVID-19 at particularly elevated risk for disease-related complications.

Under these circumstances, couples undergoing IVF treatments need to cope with the added uncertainty of the potential risks posed by COVID-19 to the safety and success of treatment and the possible implications in case of a pregnancy [22]. The aim of this double-armed, observational, cohort study is to examine the effect of COVID-19 disease on the results of IVF treatment and early pregnancy outcomes of IVF pregnancies, in order to provide evidence-based consultation to our patients.

## 2. Methods

The study contained two arms which differed according to the timing of contracting COVID-19: (1) illness before the treatment cycle, and (2) illness early in a pregnancy resulting from IVF treatment. None of the patients were vaccinated against SARS-CoV-2.

### 2.1. The First Arm: Retrospective Cohort Analysis Comparing IVF Parameters between Women Who Had COVID-19 to Those Who Did Not

This cohort included women who underwent IVF treatment at Hadassah Medical Center IVF units during the COVID-19 pandemic (March 2020–April 2021) and had RT-PCR-confirmed COVID-19 disease less than 3 months later. All patients who were over 18 and under 45 years old, and had no contraindications to proceeding with an IVF treatment cycle, were included. The study was approved by the Institutional Ethics Committee (approval number HMO-0038-21).

Since the outbreak of the epidemic, all patients in IVF units underwent mandatory RT-PCR testing before the treatment cycle; thus, we were able to distinguish between patients who were infected and those who were not.

Data on treatment outcome parameters (gonadotropin consumption, duration of stimulation, peak serum estradiol, number of retrieved oocytes, maximum endometrial thickness, fertilization rate, total number of cleavage stage embryos, and number of top-quality embryos) were retrieved from patients’ records and embryology reports. The following variables were evaluated: age, background morbidities, parity, BMI, smoking status, indication for IVF treatment, cycle type, and cycle number, as well as time from COVID-19 recovery for both partners. The definition of top-quality embryos was 6–8 cells at grade A or B at 72 h of development. In frozen–thawed cycles, endometrial thickness was the main parameter measured.

All the parameters were compared to a previous cycle performed with the same patient within 1 year of the index cycle. In cases without a previous cycle, the comparison group included non-exposed patients matched by age, indication for IVF, and treatment protocol. BMI and smoking status were compared between exposed and matched unexposed patients to control for confounding variables. We focused on the cycle parameters and embryological outcomes.

Male COVID-19 status was also documented. Sperm concentration and motility were compared in couples in which a male partner was recovering from COVID-19.

We also performed serum D-Dimer measurements for all COVID-19 recoverees. Results of 0.5 μg/mL or higher required either postponement of the treatment or the addition of low-molecular-weight heparin to the treatment protocol.

### 2.2. The Second Arm: A Prospective Cohort Analysis of Pregnancy Outcomes among IVF-Achieved Conceptions, Comparing Patients Who Contracted COVID-19 in Early Pregnancy vs. Those Who Did Not

The primary outcome measure was early pregnancy loss, defined as a miscarriage or missed abortion diagnosed after discharge from the IVF unit with an intrauterine pregnancy demonstrating fetal cardiac activity (6–7 weeks), and until 14 weeks of gestation.

Power analysis (using PEPI-for-Windows computer software http://www.brixtonhealth.com/pepi4windows.html, accessed on 1 November 2020) was conducted with the assumption of a two-fold increase in early pregnancy loss among recovered patients, while the background early pregnancy loss was defined as 25%, with a statistical power of 80% and 5% level of significance. Accordingly, the minimum sample size was determined to be 58 patients in this arm of the study (approval number HMO-0924-20). Data were collected via telephone questionnaire. All patients included in this arm tested negative for SARS-CoV-2 by PCR at the time of IVF treatment completion and confirmation of viable pregnancy. The telephone questionnaire queried confirmed diagnosis of COVID-19, pregnancy viability, pregnancy complications, and hospital admissions.

### 2.3. Statistical Analysis

Data were extracted from the patients’ electronic medical records. Missing information was retrieved directly from the patients via telephone interview. All analyses were conducted using SPSS Statistics for Windows, Version 23.0 (IBM Corp., Armonk, NY, USA). Normally distributed continuous variables, assessed with the Kolmogorov–Smirnov normality test, were reported as means and standard deviations.

Matched data were compared using a generalized estimating equation model with an interchangeable correlation matrix. A paired t-test was used to compare normally distributed matched data. Matched nonparametric data were analyzed using the Wilcoxon test. Pregnancy complication rates and proportions were compared using the Chi-squared or Fisher’s exact test for small numbers, each as appropriate. A forward conditional logistic regression model (adjusting for age, BMI, smoking exposure, male versus non-male indication for treatment, uterine malformations, and diagnosis of recurrent pregnancy loss) was used to analyze the effect of COVID-19 exposure on pregnancy loss.

Sperm parameters were compared for each patient’s samples, analyzed before and after exposure to COVID-19, using matched t-test when the data were normally distributed, and the Wilcoxon signed-rank test in cases of non-normally distributed data.

All *p*-values were tested as two-tailed and considered significant at *p* < 0.05.

## 3. Results

### 3.1. First Arm: IVF Treatment Outcomes

The cohort included 120 cycles, of which 60 were recovered patients meeting the inclusion criteria, compared to 60 non-exposed women.

Of the 60 exposed cycles of recovered patients, 41 underwent fresh controlled ovarian hyperstimulation (COH; 13 with self-comparison and 28 with matched controls). The remaining 19 patients underwent frozen–thawed cycles (13 with self-comparison and 6 with matched controls) and contributed to the endometrial lining analysis only. Five of the COH cycles were for oocyte cryopreservation treatments; all served as their own controls. The treatment outcomes comparing exposed to non-exposed patients are detailed in Table 1. Age, BMI, and smoking status were similar for the exposed and unexposed patients among self-comparison patients and those with matched controls: mean age 34.8 vs. 31.9 years (*p* = 0.06); BMI 23.9 vs. 25.43, (*p* = 0.261) for the self-comparison and matched patients, respectively. Smoking rates were 12.8% vs. 12.5%, respectively (*p* = 1). A separate analysis of the matched control cohort showed that BMI and smoking were similar between the groups (*p* = 0.8 for BMI, *p* = 0.6 for smoking status).

Treatment cycle parameters did not differ significantly between the exposed and non-exposed groups, including number of oocytes, endometrial lining thickness, fertilization rate and number of top-quality embryos (Table 1). None of the frozen embryo cycles were excluded due to insufficient endometrial lining.

Among the 38 patients who underwent embryo transfer, 17 conceived, of whom 6 (35.3%) experienced pregnancy loss. Compared to the post-discharge pregnancy loss of the non-exposed patients (33.3%), the difference was not statistically significant (*p* = 0.5). Serum D-Dimer was measured in 15 of the exposed patients. The mean serum concentration was 0.35 ± 0.08 (range 0.19–0.44) μg/mL. No thromboembolic complications were recorded in any of the exposed patients.

#### Sperm Quality

Eleven male partners also contracted COVID-19 and were within 3 months of recovery. None of the affected male partners required hospitalization. Their sperm analysis parameters during the fertilization process compared to pre-COVID-19 are detailed in Table 2. The sperm demonstrated a significant decline in concentration following COVID-19 recovery (*p* = 0.008); however, no significant change in sperm motility was apparent.

### 3.2. Second Arm: IVF Early Pregnancy Outcomes

A total of 189 IVF COVID-19 *naïve* patients, who were discharged from the clinic following the demonstration of a clinical pregnancy, were contacted. Obstetric outcomes of IVF-achieved pregnancies were compared between 30 women (15.9%) who had COVID-19 and 159 who did not (Table 3). The rate of post-discharge exposure in this group of patients was 15.9% (30 patients). None of the 30 women developed severe COVID-19 disease requiring hospitalization. The rates of non-COVID-19-related hospitalizations, pregnancy loss, and other clinically significant complications did not differ between the groups. We performed a logistic regression analysis for pregnancy loss: interestingly, the model demonstrated that a diagnosis of recurrent pregnancy loss was significantly associated with a pregnancy loss in the study cohort (odds ratio 9.8), while the other variables, including COVID-19 exposure during pregnancy, were not (see Supplementary Table S1).

## 4. Discussion

Since the emergence of SARS-CoV-2 and the COVID-19 pandemic, a plethora of data have accumulated on the virus and its clinical effects on different organ systems, age groups, and special clinical conditions. This information has allowed for the rapid development of effective vaccines and the identification of susceptible groups of patients, among them pregnant women, especially those with comorbidities [16,18].

In this study, female patients who had recovered from COVID-19 did not demonstrate compromised IVF treatment nor a higher rate of early pregnancy loss as compared to the non-exposed patients. Contrary to the initial assumption, the rate of clinical pregnancy loss did not differ. Moreover, serum D-dimer concentrations were normal among recovered patients and no thromboembolic events nor other hematological complications were observed.

Our results agree with recent reports on the outcomes of IVF treatment and early pregnancies among COVID-19 patients. Several studies have examined peak serum estradiol, oocyte yield (including mature oocytes), fertilization rate, embryo quality and clinical pregnancy rate in COVID-19 recoverees, and most found no difference between recovered and non-exposed groups [11,23,24]. These studies included only mild cases and were similar to the patients in our study. One study, however, found lower oocyte yield in the COVID-19 group, when the interval from disease to treatment cycle was at least 6 months [24], suggesting a possible long-term effect on follicle recruitment. Another study [10] found lower oocyte yield among recovered COVID-19 patients over 35 years of age, suggesting older age increases vulnerability to the effects of the virus. All the patients in the study group were asymptomatic or presented mild COVID-19 symptoms; hence, the severity of the disease was not related to the difference in the results. Interestingly, a small study from Israel showed similar IVF treatment outcomes among couples recovering from COVID-19, but with significant decreases in the number of top-quality embryos [25]. The difference may be due to the small sample size (nine couples).

In a review article by Carp-Veliscu et al. [26], four studies compared serum anti-Mullerian hormone (AMH) between people recovering from COVID-19 with different levels of severity and non-exposed controls. Three of the four reported no detectable differences in AMH. However, one study reported lower AMH levels among the COVID-19 recoverees and higher FSH levels, suggesting a smaller functional ovarian reserve. The authors note that the patients in this study presented with mild COVID-19 symptoms upon admission, and that most were treated with antiviral and even antibiotic therapy. The median age in the study group was 43 years. These characteristics might explain the difference in results compared to other studies.

Levi-Setti et al. [27] reported that the rate of pregnancy loss among IVF patients recovering from COVID-19 was not affected. Similarly, Calvo et al. [28] reported on the perinatal outcomes of 1347 pregnant women, among whom 74 conceived following IVF. This multicenter study compared the rates of early pregnancy loss, pregnancy complications, and mode of delivery according to COVID-19 disease status. The only difference found was a significant increase in the rate of cesarean sections among recovered patients. In these studies, most of the patients experienced mild COVID-19 disease.

The following studies did not necessarily include pregnancies achieved through in vitro fertilization or only early stage of pregnancy; however, they provide additional reassurance regarding outcomes related to infection during pregnancy. First is a systematic review and meta-analysis [29] that examined the risk of intrauterine fetal demise after 20 weeks of gestation and neonatal death in recovered versus non-exposed patients on admission to delivery. No significant difference was found between the groups. The authors reported that there was no significant increase in the risk of preterm birth or maternal death. A second meta-analysis compared the outcomes of over 2 million births during the pandemic to over 28 million pre-pandemic births. The study showed that pregnancies during the pandemic were associated with a lower rate of spontaneous preterm birth, with similar odds for stillbirths [30].

However, a study conducted in California that included 43,886 pregnant women, of whom 1332 had contracted SARS-CoV-2 during pregnancy, found increased risk for severe maternal morbidity, preterm birth, and venous thromboembolism, suggesting infection may be associated with increased risk for perinatal complications [31]. Most of the patients were of Hispanic origin and had underlying diseases, including autoimmune diseases, allergies, and chronic hypertension. In addition, the authors pointed out that they were unable to assess the severity of illness or symptoms associated with SARS-CoV-2 infection, although 23% of patients were hospitalized after infection while in our study none of the patients were hospitalized due to COVID-19-related morbidity.

In the current study, male partners had reduced sperm concentrations after recent recovery from COVID-19. These results are consistent with several studies that examined the effect of COVID-19 infection on sperm parameters. Three systematic reviews and meta-analyses showed a decrease in sperm concentration after exposure [32], with two also finding a decrease in sperm motility [33,34]. The clinical course of the participants’ disease in these studies was of varying degrees of severity. Another study [35], which included 41 participants with mild COVID-19 disease, showed a significant decrease in sperm concentration after exposure. However, all participants received antiviral treatment, and according to the authors, the effect of this treatment on sperm parameters is unknown.

Importantly, the compromise in sperm quality in our study did not translate into poorer IVF outcomes, possibly due to the overall treatment parameters, or because the compromise was not severe enough. Wang et al. [36] also examined the effect of COVID-19 on the sperm parameters of exposed patients, and the effect on IVF treatment outcomes. Fifty men were included in the study group. Most participants experienced asymptomatic or mild disease. No differences were found in sperm concentration and motility between the study and the control groups. In addition, the study did not find impaired IVF outcomes among the exposed group regarding implantation rate, biochemical pregnancy rate, clinical pregnancy rate, nor early miscarriage rate, with the exception of lower blastocyst formation rate post exposure to COVID-19. A possible reason for the difference in sperm concentration results is the longer recovery time from exposure in this study, as the sperm samples were taken at least four months after exposure. Since the patients in our study provided the samples within the 3-month spermatogenesis cycle, it is likely that the effects of COVID-19 on the quality of sperm were related to a transient adverse effect of the fever caused by the general illness. It is still not clear whether the decline in sperm quality remains after the effects of the general illness subside [15]. In this context, an interesting study by Guo T-H et al. [37] examined the semen of 41 participants who had contracted COVID-19, at a median of 56 days from exposure. This analysis showed a decrease in sperm concentration. Twenty-two participants agreed to provide another sample, at a median of 85 days from exposure; the sperm parameters were improved compared to the first sample. A literature review [38] found similar results and showed a decrease in sperm parameters in the range of up to 3 months from exposure to the virus, while in the range of more than 3 months from exposure no significant difference was found. Most patients in these studies experienced mild-to-moderate illness, with fever as a very common symptom. None of the patients in our study presented with symptoms suggesting direct infection of the testicles; however, the effect of a febrile illness on sperm production may have played a role. A study that compared sperm parameters of fever-positive and fever-negative male patients who had recovered from COVID-19 showed significantly lower sperm volume, concentration, and total motility in the febrile group [39]. A systematic review [33] found a correlation between the presence of fever and a decrease in sperm concentration.

Our study was limited by the small sample size, limiting subdivision according to female/male involvement, and further study of the male partner’s disease pattern and its significance is required in future larger studies. In this study, COVID-19 cases were considered mild (as none of the study cohort was hospitalized due to COVID-19-related morbidity); hence, no conclusions can be drawn about the effect of the virus in more severe cases. Sample size calculations were based on the best estimates of exposure effect available at the time of the study design. Among the study patients, the time between recovery and the onset of the treatment cycle ranged from 2 weeks to 3 months (mean 1.75 months). Thus, our results only address the early post-exposure period. We would also like to note that our study included only unvaccinated patients. The issue of vaccine safety has been extensively researched, including an emphasis on the effect on fertility. In any case, the anti-SARS-CoV-2 vaccine was not associated with adverse effects on IVF outcomes nor upon early pregnancy outcomes after IVF [11,40,41]. Generalizing the current study results to other populations may also be limited by specific treatment characteristics such as cleavage stage vs. blastocyst stage embryo transfer.

The uniqueness of this study is its comprehensive approach. We evaluated all components of IVF fertility treatments, including monitoring early clinical pregnancies. Specifically, information regarding the effect of COVID-19 on early pregnancies achieved through IVF is limited, adding further importance to this study. In addition, combining sperm analysis after exposure, together with the effect of this exposure on the results of IVF treatments, is important for a broader understanding of the effect of the virus.

Post-COVID-19 follow-up data are still being collected. Whether COVID-19 has prolonged effects on reproductive function remains an open question. Additional, larger studies are required to determine the effects of COVID-19 on embryo quality, rate of aneuploidy and long-term effects and, in particular, the effect on spermatogenesis and oocyte yield. It cannot be ruled out that new variants of SARS-CoV-2 might cause additional outbreaks, which may have different effects on the reproductive systems of men and women [42]. Therefore, the results of various studies may differ according to the variant.

To conclude: the present study did not detect any detrimental effects on IVF treatment parameters nor short-term pregnancy loss due to the SARS-CoV-2 virus. Sperm concentration was compromised post-exposure, but this did not affect treatment outcomes.

Further research is needed to expand our knowledge about the long-term effects of COVID-19 on fertility.

## Figures and Tables

**Table 1:** Cycle outcomes for post-COVID-19 IVF treatment compared to non-exposed matched cycles.

Parameter	Exposed *	Non-Exposed *	*p*-Value
	Mean ± SD, Median		
Total FSH dose (IU)	2412.63 ± 1376, 2100	2184 ± 1028.1, 2025	0.19
COH days	10.12 ± 2.3, 10	9.2 ± 2.2, 9	0.05
Maximum estradiol (pmol/L)	9044.24 ± 6341.8, 7273	8735.9 ± 5291.8, 7953	0.7
Maximum endometrial thickness, mm	10.8 ± 2.5, 0.7	10.5 ± 2.8, 10	0.8
Number ova aspirated	10.73 ± 6.9, 9	12.22 ± 9.3, 10	0.3
Number ova fertilized	6.8 ± 4.5, 6	6.7 ± 5.3, 6	0.9
Number cleavage stage embryos	6.56 ± 4.4, 6	6.0 ± 4.4, 5	0.5
Number top-quality embryos	4.57 ± 3.7, 3	3.88 ± 2.8, 3	0.3

COH = controlled ovarian hyperstimulation. * *n* = 41 matched cycles for FSH dose, COH days, maximum estradiol, aspirated ova; *n* = 36 matched cycles for fertilized *ova*, cleaved embryos and top-quality embryos; *n* = 60 matched cycles for endometrial thickness.

**Table 2:** Sperm parameters of exposed male partners: pre- and post-COVID-19 disease.

Variable (*n* = 11)	Mean ± SD	Median	*p*-Value
Concentration (mil/mL)			
Before	19.4 ± 18.3	16.5	0.008
After	12.7 ± 16.07	6.27	
Motility (%)			
Before	32.3 ± 29.7	24.5	0.2
After	22.5 ± 23.7	25.0	

SD, standard deviation. IVF outcome parameters in fresh cycles involving fertilization were also compared according to male partner disease status. Male exposure status did not affect fertilization (*p* = 0.74), number of embryos (*p* = 0.68), or top-quality embryos (*p* = 0.65).

**Table 3:** Pregnancy outcomes of COVID-19-exposed versus non-exposed IVF conceptions.

Pregnant Patients Contacted (*n* = 189)	SARS-CoV-2		*p*-Value
	Exposed (*n* = 30)	Not Exposed (*n* = 159)	
Hospitalizations (non-COVID-19 related)	2/30 (6.66%)	12/159 (7.5%)	0.63
Pregnancy complications (other than pregnancy loss)	4/30 (13.3%)	18/159 (11.3%)	0.46
ER visits	7/30 (23.3%)	24/159 (15.1%)	0.18
Pregnancy loss	7/30 (23.3%)	41/159 (25.8%)	0.48

## Data Availability

Anonymized data will be available upon request.

## Supplementary Materials

The following supporting information can be downloaded at: https://www.mdpi.com/article/10.3390/jcm11185265/s1, Table S1: Logistic regression summary model-Pregnancy loss according to COVID-19 exposure, age, BMI, diagnosis, smoking status, uterine malformations and recurrent pregnancy loss history.
