# Supplementary material for: Mild COVID-19 Was Not Associated with Impaired IVF Outcomes or Early Pregnancy Loss in IVF Patients
Source: J Clin Med. 2022 Sep 6;11(18):5265. doi: 10.3390/jcm11185265 (PMC9505769; doi:10.3390/jcm11185265)
Supplement: Supplementary file 1 [file jcm-11-05265-s001.zip › jcm-1849504-supplementary.pdf]

Supplementary Table S1. Logistic regression summary model—Pregnancy loss according to COVID-19 exposure, age, BMI, diagnosis, smoking status, uterine malformations and recurrent pregnancy loss history .

**Not in Equation**	***p*-Value**
Age	0.05
BMI	0.43
Diagnosis(male vs. non male)	0..65
Smoking	0.09
Uterine malformation	0.11
COVID-19 exposure	0.56
**In equation**	
RPL	0.01
